# Protein conjugated with aldehydes derived from lipid peroxidation as an independent parameter of the carbonyl stress in the kidney damage

**DOI:** 10.1186/1476-511X-10-201

**Published:** 2011-11-07

**Authors:** Rafael Medina-Navarro, Renato Nieto-Aguilar, Cleto Alvares-Aguilar

**Affiliations:** 1 Department of Experimental Metabolism. Center for Biomedical Research of Michoacán (CIBIMI-IMSS), Michoacán, México; 2 Department of Research in Clinical Epidemiology. Center for Biomedical Research of Michoacán (CIBIMI-IMSS), Michoacán, México; 3 Postgraduate Studies Division, Faculty of Odontology. University of Michoacán (Universidad Michoacana de San Nicolás de Hidalgo, UMSNH), Michoacán, México

**Keywords:** fatty acids lipid peroxidation, protein carbonyl, unsaturated aldehydes, renal failure, rhabdomyolysis

## Abstract

**Background:**

One of the well-defined and characterized protein modifications usually produced by oxidation is carbonylation, an irreversible non-enzymatic modification of proteins. However, carbonyl groups can be introduced into proteins by non-oxidative mechanisms. Reactive carbonyl compounds have been observed to have increased in patients with renal failure. In the present work we have described a procedure designed as aldehyde capture to calculate the protein carbonyl stress derived solely from lipid peroxidation.

**Methods:**

Acrolein-albumin adduct was prepared as standard at alkaline pH. Rat liver microsomal membranes and serum samples from patients with diabetic nephropathy were subjected to the aldehyde capture procedure and aldol-protein formation. Before alkalinization and incubation, samples were precipitated and redisolved in 6M guanidine. The absorbances of the samples were read with a spectrophotometer at 266 nm against a blank of guanidine.

**Results:**

Evidence showed abundance of unsaturated aldehydes derived from lipid peroxidation in rat liver microsomal membranes and in the serum of diabetic patients with advanced chronic kidney disease. Carbonyl protein and aldol-proteins resulted higher in the diabetic nephropathy patients (p < 0.004 and p < 0.0001 respectively).

**Conclusion:**

The aldehyde-protein adduct represents a non oxidative component of carbonyl stress, independent of the direct amino acid oxidation and could constitute a practical and novelty strategy to measure the carbonyl stress derived solely from lipid peroxidation and particularly in diabetic nephropathy patients. In addition, we are in a position to propose an alternative explanation of why alkalinization of urine attenuates rhabdomyolysis-induced renal dysfunction.

## Background

Oxidation can cause proteins to suffer loss of catalytic function, degradation, proteolysis and several degrees of denaturation [1,2]. Protein oxidation increases during aging and has been consistently associated with many pathologies such as diabetes, atherosclerosis, cataracts, renal damage, and more [3,4]. One of the well-defined and characterized protein modifications usually produced by oxidation is carbonylation, an irreversible non-enzymatic modification of proteins [5]. Therefore, oxidation of protein can be measured by the addition of carbonyl groups. The spectrophotometric assay using 2, 4-dinitrophenylhydrazine (DNPH) constitutes one of the primary ways of detecting carbonyls, since it is relatively easy, fast and inexpensive [6,7]. Carbonyl groups are introduced into proteins by metal-catalyzed oxidation and a variety of reactive nitrogen species (nitric oxide, peroxinitrite), reactive chlorine species (hypochlorous acid, nitryl chloride) and reactive oxygen species (hydrogen peroxide, hydroxyl radical, peroxiradicals, ozone, etc.)[8-10].

However, carbonyl groups can be introduced into proteins by non-oxidative mechanisms, and this can increase carbonyl levels in proteins in an extensive manner. Carbonyl groups can be introduced into proteins by reaction with molecules such as acrolein, 4-hydroxy-2-nonenal (HNE) and other alpha-beta unsaturated aldehydes derived from lipid peroxidation that react with protein residues via several kinds of reactions, included Schiff base formation and Michael addition reactions [11]. Hence, the presence of carbonyl is not necessarily indicative of direct oxidation of amino acid residues in proteins, and the importance of lipid peroxidation and the participation of reactive low molecular weight aldehydes on protein carbonylation have been demonstrated [12-14].

Reactive carbonyl compounds derived from lipids and carbohydrates by both oxidative and non-oxidative routes have been observed to have increased in patients with renal damage. These compounds react with proteins, resulting in the condition described as "carbonyl stress", which underlies the development of uremic complications [15]. Uremic plasma contains elevated levels of low weight molecular carbonyl compounds [16]. These do not seem to be secondary products of protein glycation nor do they depend on glucose plasma concentration, but more probably are reactive carbonyl compounds derived from carbohydrates, lipids and amino acids [16,17]. Rhabdomyolisis with extensive muscle release of myoglobyn (Mb) generates kidney damage caused by redox cycling of the heme radicals [18]. Lipid peroxidation catalyzed by the hemoprotein's radicals is probably the more documented explanation for the oxidative damage associated with rhabdomyolysis [19], and this condition constitutes another potential source of the alpha beta unsaturated aldehydes and carbonyl stress in the kidney.

Proteins have an enormous capability to conjugate with alpha-betha unsaturated aldehydes. At alkaline pH albumin isomerizes and exposes cysteine thiolate anions. Previous research indicates that the highly nucleophilic thiolate state is the adduct target for the alfa, beta-unsaturated carbonyl compounds [20-22]. We found that, in addition to the described reactions, unsaturated aldehydes at alkaline pH can undergo aldolic condensation linked to the proteins and form stable adducts with corresponding increases in absorbance at 266 nm. Taking advantage of properties mentioned we implement a simple procedure to calculate the abundance of these compounds from a sample containing proteins such as serum or plasma (aldehyde capture).

We observed an intense aldehyde capture during the rat liver microsomal peroxidation catalyzed by NADPH, demonstrating the participation of membranes fatty acids phospholipids as the source of alpha beta unsaturated aldehydes. Then, serum samples were subjected to the aldehyde capture and the results permitted us to corroborate the alpha-beta unsaturated aldehyde-dependent carbonyl stress derived from lipid peroxidation in the serum of diabetic patients with advanced chronic kidney disease.

## Results

### Acrolein condensation proceeds even with protein linked (aldehyde capture)

**Figure 1:**
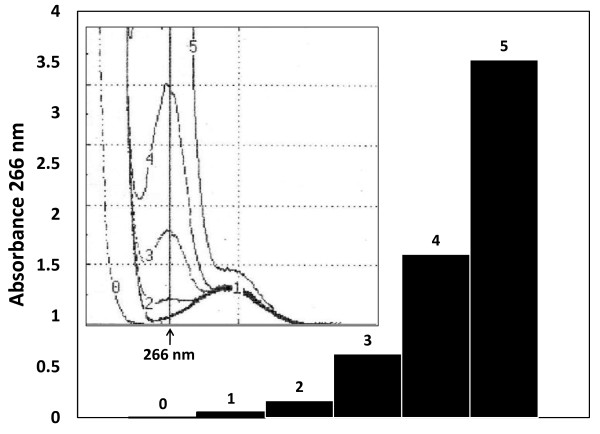
**Spectral scans and the kinetic of aldol-protein formation with respect to the time**. At the internal graph, aldehyde condensation of acrolein in the presence of albumin at alkaline pH proceeds from 0 to 50 minutes (number 0-5 on the line), making grow the absorbance denoted by ascending peaks at 266 nm. One more peak, corresponding to protein absorbance is showing around 280 nm but this time without apparent time dependent changes. At right side the bars denoting the exponential behave corresponding to the protein-aldehyde condensation kinetic. The data depicted correspond to one of the curves obtained using the described experimental conditions such as was explained in material and method section.

**Figure 2:**
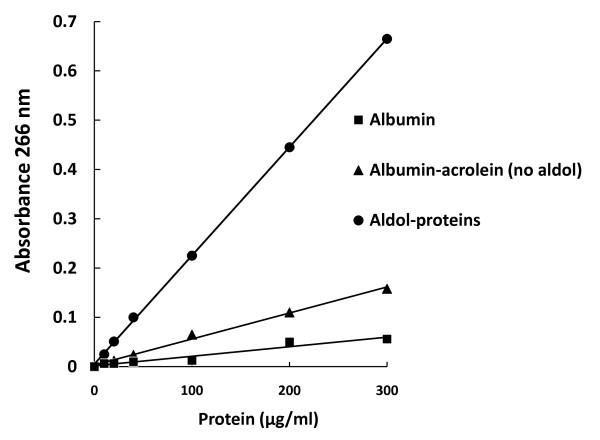
**Standard curve representing the linear relationship between the protein-acrolein adduct concentration and the absorbance at 266 nm (Aldol-Proteins)**. Parallel standard curves were made with only albumin and albumin plus acrolein without the addition of sodium hydroxide (Albumin and Albumin-acrolein no aldol). The standard curves performed in this way correspond to the values of the intrinsic absorbance of albumin (and albumin-acrolein no aldol) around the 280 nm. Samples for the standard curves were prepared as described in Material and Methods Standard preparation section. The absorbance of each aliquot was read against a blank of only guanidine hydrochloride 6M. Data depicted correspond to one of the standard curves obtained.

The kinetic of the aldehyde condensation in an alkali-catalyzed aldol reaction can be observed in Figure 1 (scan and bars 0 to 5). Acrolein in the presence of albumin polymerized (266 nm) in their bulk solution in a time dependent manner (0-50 minutes). A second independent peak can be observed at 280 nm (Figure 1 scan, line 1) and corresponds to the intrinsic absorbance of the albumin, which does not change with respect to time. The same strong absorbance of aldehyde at 266 nm in fatty acids protein-free solutions can be presented (Additional File 1) and follows a similar reaction kinetic, as been observed elsewhere [23,24]. Increasing amounts of acrolein-albumin adduct (aldol-proteins) were used to perform standard curves, one of which is presented in Figure 2. The slopes of each curve are determined by differences in absorbance at 266 nm in relation with adduct concentration. This way the average slope values of the two principal curves were 1851 and 411 for the Albumin-acrolein no aldol and the aldol-protein respectively. Some no catalyzed aldehyde captures and then spontaneous aldol-protein formation can be observed (Albumin-acrolein (no aldol) cuerve).

### Aldol-protein formation in the catalyzed microsomal peroxidation and its inhibition with bisulfite

**Figure 3:**
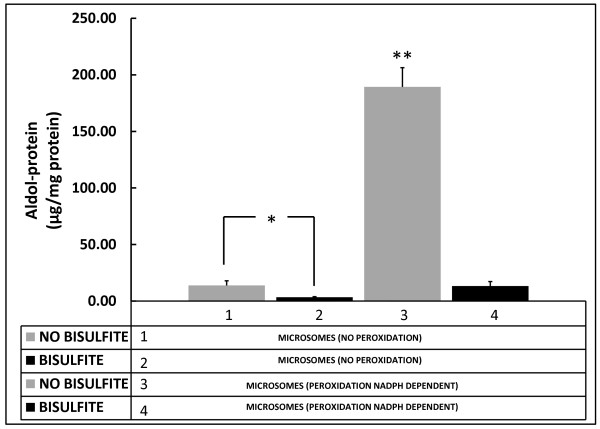
**Aldol-proteins formation in the NADPH-dependent microsomal peroxidation**. The products of catalyzed microsomal peroxidation increase the absorbance of the control sample at 266 nm by a factor of more than 14 (Bars #1 vs. #3). When sodium bisulfite was added at the end of the incubation period of peroxidation, the absorbance fell (Figure 3, #4), and reached values close to its control. Even in the control case, a fall of the initial amount could be observed with the addition of bisulfite (Bars #1 vs. #2). Bisulfite interferes directly with carbonyl groups derived from aldehydes in the aldolic condensation. * p < 0.01; **p < 0.0001 compared with all the other groups.

The products of catalyzed microsomal peroxidation increase the absorbance of the control sample at 266 nm by a factor of more than 14 (Figure 3, #1 vs. #3). Protein presented in the reaction mixture corresponds exclusively to the membrane proteins, and then the product obtained after sample precipitation corresponds to the aldol-proteins. When sodium bisulfite was added at the end of the incubation period of peroxidation, the absorbance fell (Figure 3, #4), (p < 0.0001) and reached values close to its control. Even in the control case (MICROSOMES NO PEROXIDATION), a fall of the initial amount could be observed (Figure 3 #2; p < 0.01) with the addition of bisulfite. Bisulfite interferes directly with carbonyl groups derived from aldehydes in the aldolic condensation.

### Protein carbonyl and Aldol-proteins in serum samples from patients with diabetic nephropathy

Table 1 summarizes the aldol-protein and protein carbonyl content of serum samples of 21 healthy subjects and 18 patients with diabetic nephropathy. All the patients have a GFR in the range 16-45 ml/min/1.73 m^3 ^and chronic stage 3-4 kidney disease (moderate to severe GFR reduction); meanwhile the healthy volunteers presented a GFR > 90 ml/min/1.73 m^3^, calculated from creatinine concentration and the Cockroft-Gault formula [25]. Carbonyl protein and aldol-proteins resulted higher in the diabetic nephropathy patients (p < 0.004 and p < 0.0001 respectively). Introduction of carbonyl groups to the serum sample proteins after aldehyde capture corroborated the aldehyde incorporation (p < 0.002) (A comparative graph is presented in Additional file 2).

**Table 1:** Amount of the protein carbonyls and aldol-proteins in serum samples

	Control group n = 21	Diabetic Nephropathy n = 18	p value
Carbonyl protein (nmol/mg of protein)	1.17 ± 0.32	1.74 ± 0.54	0.004
Aldol-proteins (μg/mg of protein)	106.75 ± 5.7	123.35 ± 14.9	0.0001
Carbonyl protein after aldehyde capture (nmol/mg of protein)	1.09 ± 0.17	3.24 ± 0.55	0.002
GFR* (ml/min/1.73 m^2^)	90.9 ± 5.3	30.4 ± 14.3	0.00002

*Glomerular Filtration Rate

## Discussion

In the present work we have described a procedure to calculate the protein carbonyl content derived from unsaturated aldehydes, by the use of changes experimented by the albumin molecule at alkaline pH. Formation of stable adducts or aldol-proteins in serum samples constitute a practical and novelty strategy to measure the carbonyl stress derived from lipid peroxidation.

Lipid peroxidation is one of the principal outcomes of carbonyl stress damage to biological components. The most abundant products of lipid peroxidation are aldehydes such as acrolein, malondialdehyde (MDA), 4-hydroxy-2-nonenal (HNE) and 4-hydroxy-2-hexenal (HHE), and are frequently measured as indicators of lipid peroxidation [26]. Conjugated diene measurement is useful in studies of pure lipids and it measures an early stage in the peroxidation process [27]. The measurement of malondialdehyde (MDA) by the thiobarbituric acid reactive substances assay (TBARS) is generally not a good method when applied to biological samples, although it has been widely utilized in the past [28]. These compounds and some others like isoprostanes are all measurable in biological fluids but the analytical methods used turned out to be complex and require sample preparation involving extraction and purification steps [29].

In the present work, we calculated the abundance of alpha beta unsaturated aldehydes in the plasma of diabetic patients with and without renal damage. We used a simple procedure to calculate the content of aldehydes captured by serum proteins. First we used microsomal membranes to demonstrate the aldehyde condensation-dependent absorbance increase at 266 nm and the plausible protein-dependent aldehyde capture. The aldehyde incorporation to proteins appears as independent component of carbonyl stress in serum of diabetic nephropathy patients. The aldehyde incorporation was abolished in the microsomal peroxidation NADPH-dependent through the use of sodium bisulfite, an efficient nuclephile agent that removes aldehyde groups by attacking the carbon atom of the carbonyl group. Additionally, the intense aldehyde-capture derived from the microsomal peroxidation helps us to find the source of these particular aldehydes in the unsaturated fatty acids derived from membrane phospholipids.

The oxidation of biomolecules such as carbohydrates and lipids and/or the inadequate detoxification of carbonyl compounds may contribute to the progression of complications of end-stage renal disease [30]. A better understanding of the nature of carbonyl stress in diabetic glomerular lesions would help in the search for novel approaches to confront diabetic renal damage. Additionally, if the generation of reactive carbonyl compounds is not only secondary to oxidative stress but also an active contributor to the renal disease pathogenesis, as has been suggested [31,32], an inhibition of protein carbonylation reaction might be relevant, and the approaches to measuring the aldehydes abundance might be very useful in the development of new treatment strategies.

From the results obtained we can corroborate the importance of unsaturated aldehydes in the uremic plasma as an independent parameter of carbonyl stress. In addition, we are in a position to propose an alternative explanation of why alkalinization of urine attenuates rhabdomyolysis-induced renal dysfunction [33-35]. Although one acceptable explanation is that alkalinization enhances the myoglobin (Mb) solubility [34] and that lipid peroxidation reactions are accelerated at acidic pH [35,36], a plausible alternative can be deduced from data presented here, suggesting that alpha-beta-unsaturated compounds condensation catalyzed at alkaline pH could reduce renal dysfunctions during rabdomyolisis. It is possible to suppose that polymerization of reactive aldehydes represents a way to block carbonyl groups in large amounts and promote carbonyl stress amelioration.

## Materials and methods

Unless noted otherwise, biochemicals were obtained from Sigma Chemical Co., St. Louis, MO; albumin from bovine serum (BSA) (fraction V, lyophilized powder 98%); albumin from human serum (lyophilized powder 99%); 2, 4-dinitrophenylhydrazine (DNPH); ethylenediaminetetraacetic acid (EDTA); guanidine hydrochloride; acrolein (2-propenal) 99%, sodium bisulfite (sodium hydrogensulfite ≥ 99%) and sodium hydroxide (Sigma Ultra 98%); nicotinamide; adenosine 5'- diphosphate sodium salt (ADP), ferric Chloride (FeCl_3_) and β-Nicotinamide adenine dinucleotide phosphate reduced form (β-NADPH).

### Samples

Serum aliquots of diabetic patients of the Unit of Nephrology at General Hospital # 80 of the Mexican Institute of Social Security, Morelia, Michoacán, México were collected from samples that were used in the periodic clinical examinations. Control serum samples were collected from individuals recruited for blood donations. All patients received and gave both oral and written informed consent for the use of the aliquot samples. Blood samples were obtained in the morning after fasting by venipuncture in tubes without anticoagulant. Serum (21 controls and 18 diabetic with nephropathy) was stored at -30°C and in all cases was used 3 days after being collected. *Animal samples*. Hepatic tissue from Sprague Dowley male rats weighing 200-250 g was used. Animal procedures and management were made in accordance with the National Institute of Health Guide for the Care and Use of Laboratory Animals and approved by the Ethics Committee of the Mexican Institute of Social Security.

### Acrolein capture by albumin and protein adducts (Aldol-proteins) measurement

Standard preparation. An adduct acrolein-albumin standard curve was prepared as follows: 5 mg of human or bovine serum albumin was dissolved completely in 5 ml PBS and then incubated at room temperature with acrolein (10 μM final concentration), 100 μl of 0.01 mM EDTA and 100 μl of a 1% sodium hydroxide solution. Aldolic condensates were formed this way in a time dependent manner (Figure 1). The reaction mixture was neutralized with HCL (2N) and dialyzed overnight against PBS at 4°C. Aliquots containing increasing amounts of protein were precipitated using 30% ZnSO_4 _and centrifuged 15 minutes at 5, 000 g; the pellets were dissolved with 6M guanidine hydrochloride and incubated at 37°C for 2 hours with occasional vortex mixing. The absorbance of each aliquot was read with a spectrophotometer at 266 nm (aldolic condensation) against a blank of only guanidine hydrochloride 6M. The standard curve performed represents the linear relationship between the protein-acrolein adducts concentration or aldol-protein abundance and the absorbance at 266 nm (Figure 2, aldol-protein). Different alpha-beta unsaturated incubated with albumin and aldehydes in protein-free solutions present strong absorbance at 266 nm, although acrolein showed the maximal efficiency in adduct formation [27,28].

Parallel standard curves were made in the same conditions with only albumin and albumin plus acrolein without the addition of sodium hydroxide (Figure 2, albumin and albumin plus acrolein). The standard curves performed in this way correspond to the indirect absorbance of albumin (and albumin plus acrolein) at 266 nm, as a consequence of its intrinsic absorbance at 280 nm.

Sample procedure. Serum samples (15 μl) dissolved in 50 μl of PBS, 25 μl of 0.1 mM EDTA and 50 μl of 1% sodium hydroxide were incubated for 20 minutes at 37°C. At the end of incubation, samples were precipitated with 50 μl of ZnSO_4 _30%, dissolved with an additional 500 μl of PBS and centrifuged for 15 minutes at 5000 g. Pellets were washed two more times with 0.5 ml of PBS and finally dissolved with 6M guanidine hydrochloride and incubated at 37°C for 2 hours with occasional vortex mixing. The absorbances of the samples were read with a spectrophotometer at 266 nm against a blank of only guanidine hydrochloride 6M, as explained previously.

Protein concentration was determined by the Bradford reagent and then the concentration used of each sample was adjusted with PBS. To the final concentration of protein in guanidine, standard curves of Bradford procedure were carried out substituting the vehicle by guanidine 2M and taking aliquot of 10 μl of protein guanidine samples.

Using the absorbances of 266 nm and the slopes of the standard curves described previously (Figure 2) the Aldol-Protein concentration can be calculated as follows:

Aldol-Protein = (Abs 266 nm - F) (N2), where F is the calculated absorbance of albumin + acrolein mix (no aldol) at 266 nm and N2 corresponds to the slope of the aldol-protein adduct standard curve.

F = (Abs 266 nm) (N1) - (Abs 266 nm) (N2)/(N1), where N1 and N2 are the slopes of the standard curves of albumin + acrolein mix (no aldol) and aldol-protein respectively (1851 and 411 for the standard curves presented in Figure 2)

### Microsomal peroxidation catalyzed by NADPH

Microsomes. Hepatic tissue from Sprague Dowley rats was homogenized in 25 M sucrose. The homogenate was placed in a centrifuge tube and an equivalent volume of 0.34 M sucrose was layered underneath. The discontinuous gradient thus formed was centrifuged for 10 minutes at 700 × g, and before eliminating the crude nuclei, the supernatant was centrifuged 10 minutes at 15, 000 × g. The precipitate (crude mitochondrial fraction) was discarded and the supernatant centrifuged at 105, 000 × g for 60 minutes. The crude microsomal fraction resultant was re-suspended in KCL 125 mM (1 ml of solution/g of liver used for the preparation) for a concentration of 12.0 mg of microsomal protein/mL).

Microsomal Peroxidation. The induction of lipid peroxidation on microsomal membranes was carried out with a controlled system described previously [37,38] and with a reaction mix containing 20 mM nicotinamide, 2 mM ADP, 0.12 mM FeCl_3_, and 0.4 mM NADPH in a 80 mM KCl solution. The control samples without peroxidation were prepared as previously described but without the addition of NADPH (Figure 3, MICROSOMES NO PEROXIDATION). In the experimental procedure to inhibit lipid peroxidation by blocking the carbonyl groups, sodium bisulfite (0.01 μM) was added to the reaction mix at the end of the incubation time (1 h). The pH of the samples had been previously neutralized.

### Protein Carbonyl Measurement

Protein carbonyl determination was carried out in accordance with previous reports [39]. Protein samples containing 6 mg/ml of protein were re-suspended in 1 ml of 10 mM 2, 4-dinitrophenylhydrazine (DNPH) in 2 M HCl. The samples were incubated at laboratory temperature in the dark for 60 min, stirring at 15-minute intervals. The samples that had been previously precipitated with trichloroacetic acid (20%), centrifuged at 11, 000 × g for 10 minutes and re-suspended with a pulse of sonication, were washed three times with 1 mL of ethanol-ethyl acetate (1:1; v/v) to remove the residual DNPH reagent. The final precipitates were dissolved in 6 N guanidine hydrochloride solution (1 mL). The protein carbonyl content was determined by measuring the absorbance of the protein-2, 4 dinitrophenylhydrazone derivatives at 375 nm, using a molar absorption coefficient of 22, 000 M^-1 ^cm^-1^.

### Statistical Analysis

Results were expressed as mean ± standard deviation (SD). Groups were compared using one way ANOVA test followed by Dunet's test.

## Competing interests

The authors declare that they have no competing interests.

## Authors' contributions

RMN conceived, designed and coordinated the work, as well as prepared the manuscript. RNA participated in the co-design of the work as well as the draft of the manuscript. CAA carried out analytical and statistical analysis and participated in the co-design of the work. All authors read and approved the final manuscript.

## Supplementary Material

Additional file 1 **Aldehyde condensation from unsaturated fatty acids and in absence of proteins**. Unsaturated aldehydes from the free fatty acid (FFA) lipid peroxidation can be condensed at alkaline pH and produce a strong absorbance at 266 nm. Ferric ions (Fe) and glucose (GLU) autoxidation catalyzed by traces of transition metals are the most probable source of lipid peroxidation (references 23 and 24) and aldehyde production in a time dependent slow rate (24 hours) reaction. The maximal aldol-protein production was obtained with the additive effect of GLU and ferric ions (FFAs + Glu + Fe^3+^), but even with the use of a nitrogen environment some amount of autoxidation of FFAs generates unsaturated aldehydes and aldolic condensation such as can be observed in the left short bar. Click here for file

Additional file 2 **Validated carbonyl proteins introduction to the serum samples derived from aldehyde capture**. The differences in carbonyl content of the serum samples before and after aldehyde capture treatment were corroborated. The results demonstrated the incorporation of the carbonyl groups to the serum proteins and aldol-protein formation from aldehyde capture (p = 0.002). Only the samples of patients with diabetic nephropathy and higher level of aldol proteins presented at the same time higher levels of protein carbonyls. Results and samples correspond to the data presented in the text in Table 1. Click here for file
