# Supplementary figures and images for: Protein conjugated with aldehydes derived from lipid peroxidation as an independent parameter of the carbonyl stress in the kidney damage
Source: Lipids Health Dis. 2011 Nov 7;10:201. doi: 10.1186/1476-511X-10-201 (PMC3248915; doi:10.1186/1476-511X-10-201)

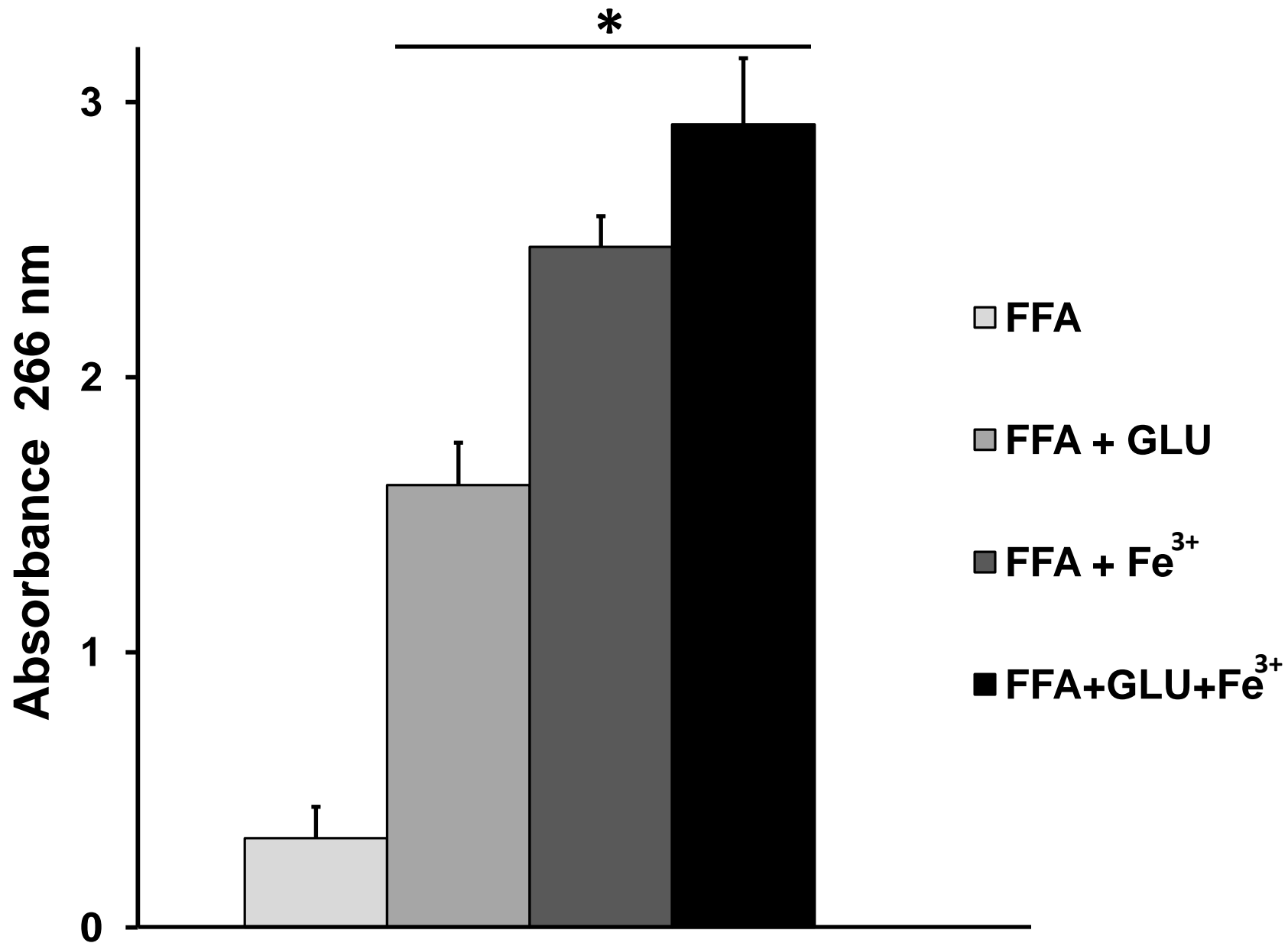

Supplement: Additional file 1 — Aldehyde condensation from unsaturated fatty acids and in absence of proteins. Unsaturated aldehydes from the free fatty acid (FFA) lipid peroxidation can be condensed at alkaline pH and produce a strong absorbance at 266 nm. Ferric ions (Fe) and glucose (GLU) autoxidation catalyzed by traces of transition metals are the most probable source of lipid peroxidation (references 23 and 24) and aldehyde production in a time dependent slow rate (24 hours) reaction. The maximal aldol-protein production was obtained with the additive effect of GLU and ferric ions (FFAs + Glu + Fe3+), but even with the use of a nitrogen environment some amount of autoxidation of FFAs generates unsaturated aldehydes and aldolic condensation such as can be observed in the left short bar. [file 1476-511X-10-201-S1.PDF]

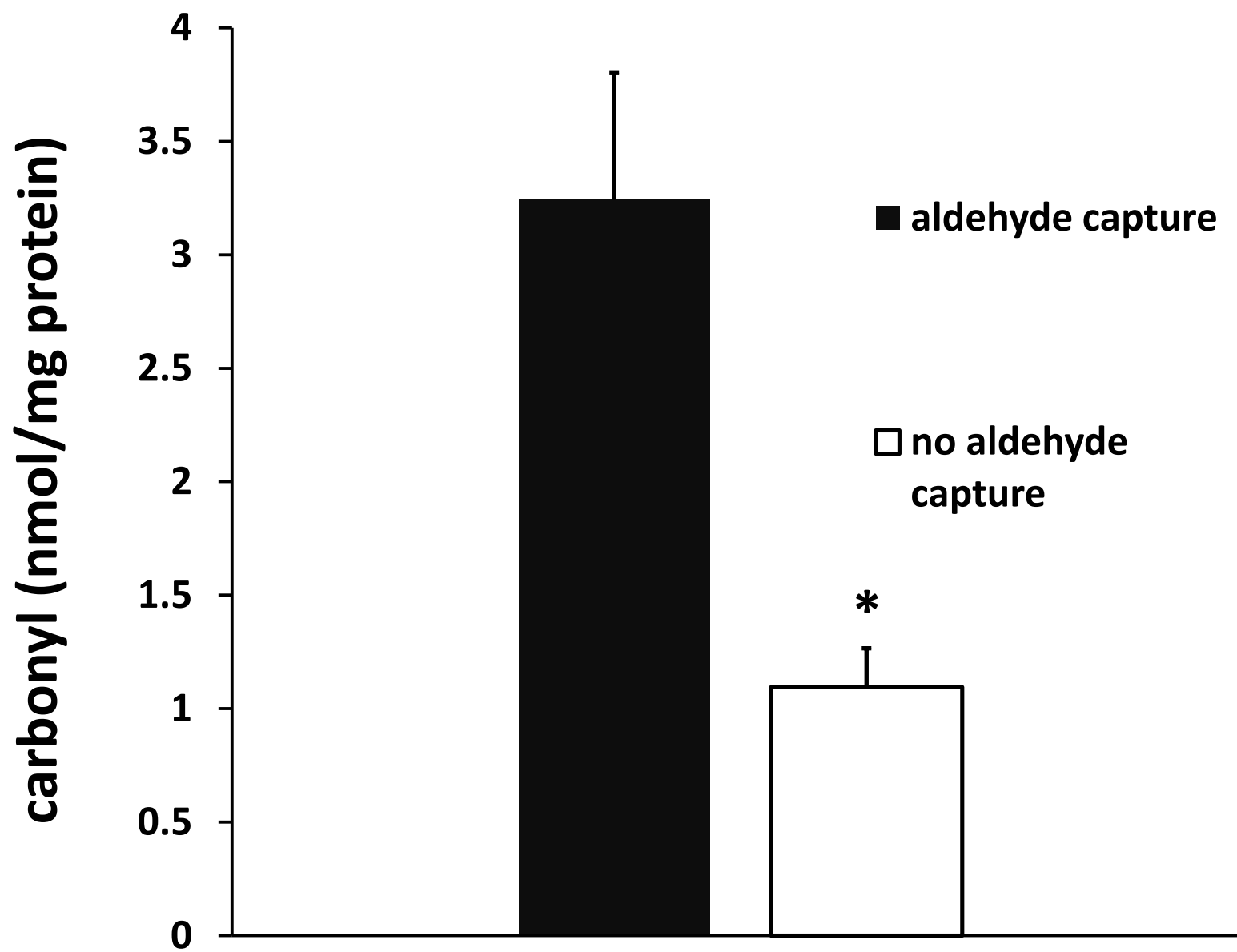

Supplement: Additional file 2 — Validated carbonyl proteins introduction to the serum samples derived from aldehyde capture. The differences in carbonyl content of the serum samples before and after aldehyde capture treatment were corroborated. The results demonstrated the incorporation of the carbonyl groups to the serum proteins and aldol-protein formation from aldehyde capture (p = 0.002). Only the samples of patients with diabetic nephropathy and higher level of aldol proteins presented at the same time higher levels of protein carbonyls. Results and samples correspond to the data presented in the text in Table 1. [file 1476-511X-10-201-S2.PDF]
